# Prevalence, risk, and outcome of deep vein thrombosis in acute respiratory distress syndrome

**DOI:** 10.1186/s12959-021-00325-3

**Published:** 2021-10-13

**Authors:** Na Cui, Chunguo Jiang, Hairong Chen, Liming Zhang, Xiaokai Feng

**Affiliations:** 1 Department of Pulmonary and Critical Care Medicine, Beijing Chao-Yang Hospital, Capital Medical University, No. 8, Gongti South Road, Chaoyang District, Beijing, 100020 People’s Republic of China; 2 Beijing Institute of Respiratory Medicine, Beijing, 100020 People’s Republic of China; 3 Department of Intensive Care Unit, Shandong Provincial Qianfoshan Hospital, The First Affiliated Hospital of Shandong First Medical University, Ji’nan, People’s Republic of China

**Keywords:** Acute respiratory distress syndrome, Caprini score, Deep vein thrombosis, Invasive mechanical ventilation, Padua prediction score

## Abstract

**Background:**

Few data exist on deep vein thrombosis (DVT) in patients with acute respiratory distress syndrome (ARDS), a group of heterogeneous diseases characterized by acute hypoxemia.

**Study design and methods:**

We retrospectively enrolled 225 adults with ARDS admitted to the Beijing Chao-Yang Hospital and the First Affiliated Hospital of Shandong First Medical University between 1 January 2015 and 30 June 2020. We analyzed clinical, laboratory, and echocardiography data for groups with and without DVT and for direct (pulmonary) and indirect (extrapulmonary) ARDS subgroups.

**Results:**

Ninety (40.0%) patients developed DVT. Compared with the non-DVT group, patients with DVT were older, had lower serum creatinine levels, lower partial pressure of arterial oxygen/fraction of inspired oxygen, higher serum procalcitonin levels, higher Padua prediction scores, and higher proportions of sedation and invasive mechanical ventilation (IMV). Multivariate analysis showed an association between age, serum creatinine level, IMV, and DVT in the ARDS cohort. The sensitivity and specificity of corresponding receiver operating characteristic curves were not inferior to those of the Padua prediction score and the Caprini score for screening for DVT in the three ARDS cohorts. Patients with DVT had a significantly lower survival rate than those without DVT in the overall ARDS cohort and in the groups with direct and indirect ARDS.

**Conclusions:**

The prevalence of DVT is high in patients with ARDS. The risk factors for DVT are age, serum creatinine level, and IMV. DVT is associated with decreased survival in patients with ARDS.

## Introduction

Deep vein thrombosis (DVT) and pulmonary embolism (PE), collectively referred to as venous thromboembolism (VTE), constitute a major global burden of disease [1]. Some studies demonstrated an increased risk of VTE in patients in the intensive care unit (ICU) [2–4]. Patients with acute respiratory distress syndrome (ARDS) are at high risk for DVT because they are susceptible both to general risk factors for VTE and to those specific to the critically ill, such as advanced age, sedation, immobilization, insertion of a central venous catheter, and mechanical ventilation (MV), combined with a severe inflammatory response and hypercoagulable states [2–6]. ARDS remains under-recognized clinically; however, therapies are limited, complications are frequent, and mortality remains significantly high [7–11]. Patients with ARDS are a heterogeneous group with significant variability in clinical presentation and outcomes. One approach to reducing these heterogeneities is to subclassify patients with ARDS as having direct (pulmonary) or indirect (extrapulmonary) ARDS based on variabilities in the pathological, radiological, and respiratory mechanical responses to different management strategies [12–19].

The incidence of DVT in patients with direct and indirect ARDS has not been investigated.

We performed a multi-institutional study to identify the prevalence, risk factors, and prognosis of DVT and to determine whether the predictors of DVT differed between direct and indirect ARDS in a cohort of patients identified with ARDS.

## Methods

### Study design and population

**Fig. 1:**
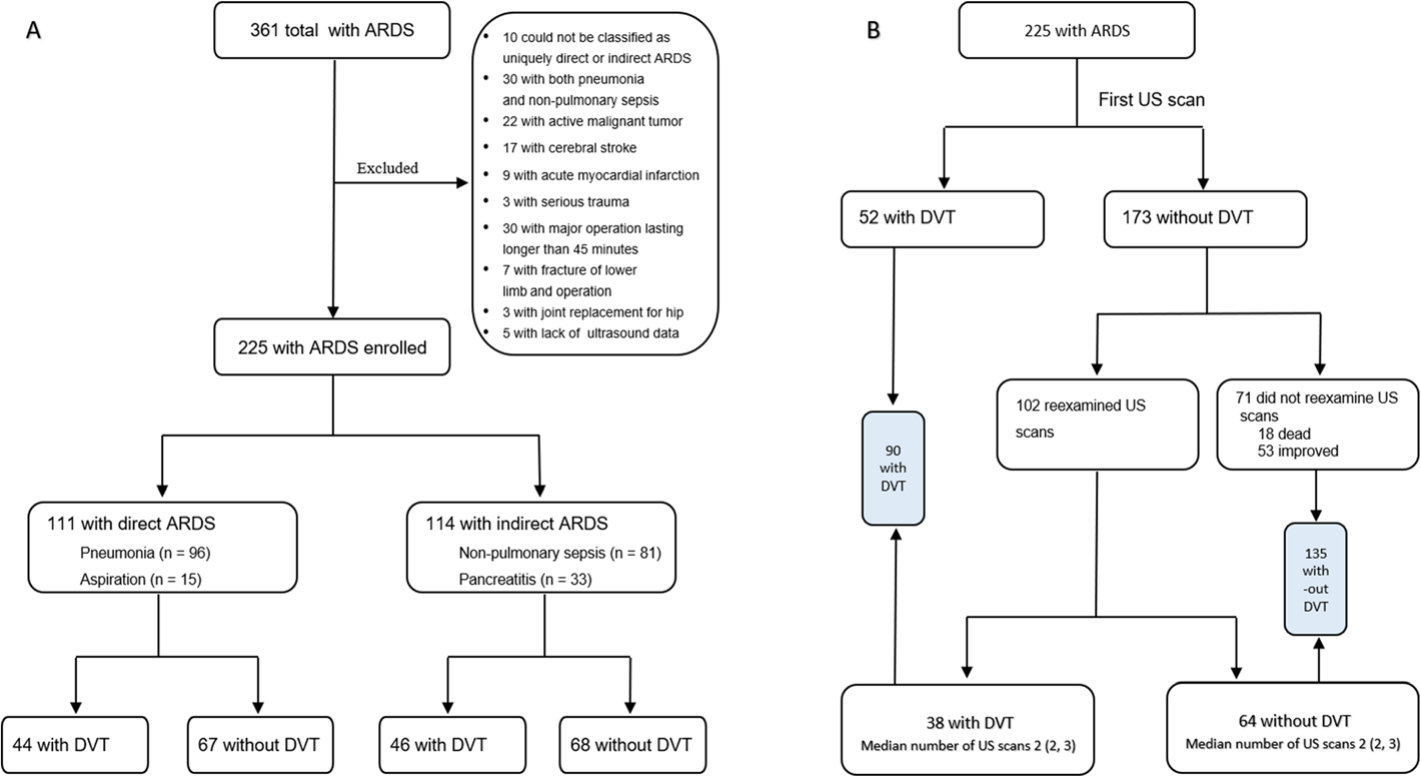
**A**, **B**, Study flow chart. **A**, flow chart for including patients; **B**, flow chart for screening for DVT. The interval from the diagnosis of ARDS to the occurrence of DVT in the DVT group was 5 (2, 9) days, and the interval from the diagnosis of ARDS to the last ultrasound examination in the non-DVT group was 5 (2, 11) days. There were no differences between the two groups (*P* = 0.784). Abbreviations: ARDS, acute respiratory distress syndrome; DVT, deep vein thrombosis; US, ultrasound

We retrospectively enrolled adult patients (≥ 18 years old) with ARDS (according to the Berlin definition) [8] who were admitted to the Department of Pulmonary and Critical Care Medicine, Beijing Chao-Yang Hospital and the Intensive Care Unit, the First Affiliated Hospital of Shandong First Medical University, from 1 January 2015 to 30 June 2020. All patients were included consecutively. Patients with ARDS were classified as having direct ARDS or indirect ARDS based on the underlying risk factors for ARDS recorded by study personnel. Patients with pneumonia and aspiration as risk factors and those with pulmonary sepsis were assigned to the direct ARDS group, whereas those with pancreatitis or non-pulmonary sepsis were assigned to the indirect ARDS group. Patients who could not be classified as uniquely direct or indirect ARDS and patients with both pneumonia and non-pulmonary sepsis were excluded. Other exclusion criteria include: active malignant tumor, cerebral stroke, acute myocardial infarction, serious trauma, major operation lasting longer than 45 min, fracture of lower limb, joint replacement for hip or knee, and lack of lower extremity venous compression ultrasound data. The first ultrasound examination was performed within 1–3 days after the diagnosis of ARDS, and then the ultrasound scan was reexamined again according to the patient’s condition. After intensive treatment, if the patient remained unstable because of conditions such as unexplained hypoxemia or cardiac insufficiency, he or she should be reexamined by ultrasound. If there was more than one ultrasound scan for a single patient, all the results were recorded. Patients were divided into a DVT and a non-DVT group according to the results of the venous ultrasound scans. The flow chart is shown in Fig. 1 (A, B).

The study was approved by the ethics committees of the Beijing Chao-Yang Hospital (2020-ke-429) and the First Affiliated Hospital of Shandong First Medical University (S003) and was conducted in accordance with the 1964 Helsinki Declaration and its later amendments or comparable ethical standards.

### Clinical data

We analyzed the medical records of the enrolled patients. Data, which included demographic information, clinical history, vital signs, laboratory findings, treatments, complications, and outcomes of the patients during hospitalization, were collected and analyzed. We analyzed the survival rates of all patients within 28 days after a diagnosis of ARDS. For the patients discharged within 28 days, we followed up by telephone concerning their survival status after discharge.

### Ultrasound assessment

Bedside ultrasound examinations were performed using a portable color ultrasound scanner (CX50, Philips Medical Systems, the Netherlands, equipped with an L12–3/S5–1 probe). The lower extremity venous compression ultrasound and echocardiographic data were obtained from the institution’s Picture Archiving and Communication System. The levels of DVT included the bilateral common femoral, deep and superficial femoral veins, the popliteal veins, and the anterior tibial, posterior tibial, peroneal, and calf muscle veins. Left ventricular and right ventricular function parameters were captured. The presence of pulmonary artery hypertension was evaluated by adding a tricuspid regurgitation pressure gradient to the estimated right atrial pressure [20].

### Definitions

ARDS was defined according to the Berlin definition [8]. Sepsis was defined according to the Third International Consensus Definitions for Sepsis and Septic Shock [21]. A distal thrombosis was defined as a thrombosis in the veins of the calf muscle or in at least 1 branch of the 3 pairs of deep calf veins (anterior tibial vein, posterior tibial vein, or peroneal vein); a proximal thrombosis was defined as a thrombosis in the popliteal vein or above. The Caprini score was defined according to the updated Caprini Risk Assessment Model (2013 Version) [22]. The Padua prediction score was defined according to the Barbar model [23]. We applied the Acute Physiology and Chronic Health Evaluation (APACHE) II score and the Sequential Organ Failure Assessment (SOFA) score to assess the severity of disease [21, 24, 25].

### Statistical analyses

Categorical variables were described as number and percentage (%) and continuous variables, as mean, standard deviation, median, and interquartile range. The Shapiro-Wilk test was used to verify normality. Differences between the DVT and the non-DVT groups were assessed by a two-sample *t-*test for normally distributed continuous variables, the Mann-Whitney U test for non-normally distributed continuous variables, and the χ^2^ or Fisher exact test for categorical variables. To determine risk factors for DVT, multivariable logistic regression analysis which was based on the factors with significant differences between DVT and non-DVT groups in univariate analysis and the factors that may be related to dependent variables from the perspective of professional knowledge was performed on the direct and the indirect ARDS subgroups. The adjusted odds ratio (OR) with 95% confidence intervals (CI) was reported. To further evaluate the observed differences in risk factors for DVT between direct and indirect ARDS, we utilized interaction terms between ARDS type and each risk factor. A receiver operating characteristic (ROC) analysis was performed to calculate the sensitivity and specificity of risk factors for screening for DVT. The comparison methods of diagnostic accuracy for screening for DVT of different ROCs in three ARDS cohorts are as follows: Patients were split by generating random numbers to produce a training data set (n*0.7) and a validation data set (n*0.3) in the overall, direct, and indirect groups respectively. The area under receiver operating characteristic curves (ROC-AUCs) for different risk factors were compared using the method of DeLong et al. [26]. Survival curves were plotted using the Kaplan-Meier method and compared between patients with or without DVT using the log-rank test. To further explore the incidence rate of DVT in patients with ARDS in ICU, we selected non-ARDS patients in ICU consecutively during the same period as controls. And then, we took death as the competitive risk and plotted 28-day cumulative incidence curves (and points estimates with 95% CI) for ARDS and non-ARDS patients. Fine-Gray test was used to compare the incidence rate of DVT between the two groups. All statistical analyses were performed using the Statistical Analysis System, version 9.4 (SAS Institute, Cary, NC, USA). All tests were two-tailed; *P* <  0.05 was considered statistically significant.

## Results

A total of 225 patients with ARDS were enrolled in this study; 111 patients were considered to belong in the direct ARDS group and 114 patients in the indirect ARDS group. The flow chart is shown in Fig. 1 (A, B).

### Ultrasound scan for screening for DVT

Lower extremity venous ultrasound scanning was performed whenever feasible for 225 patients regardless of clinical symptoms of the lower limbs (Fig. 1B), and the median number of ultrasound examinations was 1 (range, 1–5). Fifty-two (52/225) developed DVT was found and the other 173 was a negative result at the first ultrasound scan. Subsequently, 102 patients underwent more than one ultrasound scan, for whom 38 developed DVT and 64 had no DVT with 2 (range, 2–5) ultrasound examinations. The interval from the diagnosis of ARDS to the occurrence of DVT in the 38 developed DVT group was 7 (4, 14) days, and the interval from the diagnosis of ARDS to the last ultrasound examination in the 64 non-DVT group was 10 (4, 16) days. There was no difference between the two groups (*P* = 0.542). Finally, of the 225 patients, 90 (40.0%) developed DVT, including 7 with proximal DVT and 83 with distal DVT, 73 of whom had muscular calf vein thrombosis only. The incidence of asymptomatic DVT was 75 (33.3%) including 2 (0.9%) proximal DVT and 73 (32.4%) distal DVT, of whom muscular calf vein thrombosis accounted for 70 (31.1%). For all the 225 patients, the interval from the diagnosis of ARDS to the occurrence of DVT in DVT group was 5 (2, 9) days, and the interval from the diagnosis of ARDS to the last ultrasound examination in non-DVT group was 5 (2, 11) days. There was no difference between the two groups (*P* = 0.784). Five patients were clinically suspected of having PE; three were further confirmed by computed tomography pulmonary angiography (CTPA) examination. There was no difference in the prevalence of DVT in patients with direct and indirect ARDS (39.6% [44/111] vs 40.4% [46/114], respectively; *P* = 0.913; Table 1).

**Table 1:** Demographic and Clinical Characteristics of Patients with Direct and Indirect ARDS

Characteristic	Total (*N* = 225)	Direct ARDS (*N* = 111)	Indirect ARDS (*N* = 114)	*P* value
Age (years)	66 ± 17	64 ± 15	67 ± 18	0.192
Male, n (%)	144 (64.0)	84 (75.7)	60 (52.6)	< 0.001
BMI	24.1 (21.6, 26.8)	24.0 (21.0, 26.2)	24.4 (21.9, 27.0)	0.274
Bedridden time (days)	8 (4, 15)	9 (5, 15)	8 (4, 16)	0.400
APACHE II score	23 (19, 28)	22 (17, 27)	25 (19, 31)	0.010
SOFA score	8 (5, 10)	6 (4, 6)	9 (6, 11)	< 0.001
Laboratory data				
White blood cells (×10^9^/L)	16.9 (12.0, 21.5)	14.3 (10.1, 20.3)	18.2 (14.4, 23.5)	< 0.001
Neutrophils (×10^9^/L)	14.5 (10.4, 19.7)	12.8 (9.0, 17.7)	16.1 (12.6, 20.8)	< 0.001
Platelets (×10^9^/L)	183.0 (101.0, 253.5)	190.0 (120.0, 270.0)	167.0 (73.8, 238.0)	0.094
C-reactive protein (mg/L)	120.0 (89.4, 120.0)	120.0 (85.0, 120.0)	120.0 (92.2, 120.0)	0.391
Procalcitonin (ng/mL)	5.6 (2.0, 16.2)	4.1 (1.5, 13.5)	7.3 (3.0, 21.2)	0.008
Serum creatinine (μmol/L)	116.7 (66.6, 209.5)	90.5 (65.0, 193.0)	136.9 (67.9, 248.8)	0.019
D-dimer (μg/ml)	1.9 (0.9, 3.8)	1.3 (0.6, 2.5)	2.4 (1.3, 5.3)	< 0.001
PaO_2_/FiO_2_	158 (103, 199)	136 (80, 186)	170 (130, 208)	< 0.001^*^
Mild, n (%)	54 (24.0)	22 (19.8)	32 (28.1)	< 0.001^#^
Moderate, n (%)	116 (51.6)	49 (44.1)	67 (58.8)	
Severe, n (%)	55 (24.4)	40 (36.0)	15 (13.2)	
DVT, n (%)	90 (40.0%)	44 (39.6)	46 (40.4)	0.913
ICU length of stay (days)	11 (6, 24)	13 (7, 25)	10 (5, 24)	0.103
Hospital length of stay (days)	19 (12, 32)	17 (10, 29)	22 (13, 34)	0.055
Mortality, n (%)	77 (34.2)	36 (32.4)	41 (36.0)	0.577

Data are mean ± SD, median (IQR) or n (%). *P* values comparing Direct and Indirect ARDS groups were from a two-sample *t-*test, Mann- Whitney *U* test, or χ^2^ test. *P* <  0.05 was considered statistically significant

^*^χ^2^ test comparing Direct and Indirect ARDS groups

^#^χ^2^ test comparing all subcategories

*Abbreviations*: *APACHE* Acute Physiology and Chronic Health Evaluation, *ARDS* acute respiratory distress syndrome, *BMI* body mass index, *DVT* deep venous thrombosis, *FiO*_2_, fraction of inspired oxygen, *ICU* intensive care unit, *IQR* interquartile range, *mild* 200 mmHg<PaO_2_/FiO_2_ ≤ 300 mmHg, *moderate* 100 mmHg<PaO_2_/FiO_2_ ≤ 200 mmHg, *PaO*_2_ partial pressure of arterial oxygen, *SD* standard deviation, *severe* PaO_2_/FiO_2_ ≤ 100 mmHg, *SOFA* Sequential Organ Failure Assessment

### Demographic and clinical characteristics of patients with direct and indirect ARDS

Compared with the direct ARDS group (Table 1), patients with indirect ARDS had higher APACHE II scores (*P* = 0.010), higher SOFA scores (*P* <  0.001), higher white blood cell counts (*P* <  0.001), higher neutrophil counts (*P* <  0.001), higher levels of procalcitonin (*P* = 0.008), higher levels of serum creatinine (*P* = 0.019), and higher levels of D-dimer (*P* <  0.001). There were more men in the direct ARDS group (*P* <  0.001). Patients with direct ARDS had lower PaO_2_/FiO_2_ than those with indirect ARDS (*P* <  0.001).

### Demographic and clinical characteristics of DVT vs non-DVT patients in overall ARDS cohort

Compared with the non-DVT group (Table 2), patients with DVT were older (*P* <  0.001) and had lower levels of serum creatinine (*P* = 0.007), lower levels of partial pressure of arterial oxygen/fraction of inspired oxygen (PaO_2_/FiO_2_; *P* = 0.002), higher levels of serum procalcitonin (PCT; *P* <  0.001), higher Padua prediction scores (*P* = 0.023), and a higher proportion of patients given sedative therapy (*P* = 0.001) and invasive mechanical ventilation (IMV; *P* <  0.001). Patients with DVT had more deaths within 28 days after ARDS than those without DVT (*P* <  0.001). There were no differences in comorbidities (*P >* 0.05 for all; data are not shown) between the DVT and the non-DVT groups. All patients were bedridden for more than 3 days with no difference between the DVT and the non-DVT groups (*P* = 0.216). For the 135 (60.0%) patients who received VTE prophylaxis, the incidence of DVT was 37.0% (50/135); however, for the patients who did not receive VTE prophylaxis, it was 44.4% (40/90), and there was no significant difference between the two groups (*P* = 0.271). For the 108 (48.0%) patients who received low molecular weight heparin (LMWH), the incidence of DVT was 36.1% (39/108), and for the 75 (33.5%) patients who received combined treatment with LMWH and physical prevention, it was 37.3% (28/75). There was no significant difference between the two groups (*P* = 0.866). Among the 90 patients who did not receive VTE prophylaxis, 32 had anticoagulant therapy contraindications, such as stress ulcers and gastrointestinal bleeding (17 patients), platelet counts less than 50 × 10^9^/L (13 patients), and haemoptysis (2 patients). The remaining 58 (25.8%) patients had no clear high-risk factors for bleeding but did not receive VTE prophylaxis due to patient preference of non-adherence to guidelines at that time in this retrospective observational study. All the 90 patients with DVT were treated with LMWH (66 received dalteparin 5000 IU once per 12 h, 24 received nadroparin 0.1 ml/10 kg once per 12 h).

**Table 2:** Demographic and Clinical Characteristics of DVT Vs Non-DVT Patients in Overall ARDS Cohort

Characteristic	Total	DVT	Non-DVT	*P* value
	(*N* = 225)	(*N* = 90)	(*N* = 135)	
Age (years)	66 ± 17	70 ± 13	63 ± 18	< 0.001
Male, n (%)	144 (64.0)	52 (57.8)	92 (68.1)	0.112
BMI	24.1 (21.6, 26.8)	24.0 (20.8, 26.0)	24.2 (22.0, 27.2)	0.146
Direct ARDS				
Pneumonia	96 (86.5)	40 (90.9)	56 (83.6)	0.412
Aspiration	15 (13.5)	4 (9.1)	11 (16.4)	
Indirect ARDS				
Non-pulmonary sepsis	81 (71.1)	33 (71.7)	48 (70.6)	0.894
Pancreatitis	33 (28.9)	13 (28.3)	20 (29.4)	
Bedridden time (days)	8 (4, 15)	10 (5, 18)	7 (4, 15)	0.216
Caprini score	7 (5, 9)	7 (5, 10)	7 (5, 9)	0.135
Padua prediction score	6 (5, 6)	6 (5, 8)	5 (5, 6)	0.023
APACHE II score	23 (19, 28)	24 (20, 28)	23 (18, 29)	0.596
SOFA score	8 (5, 10)	7 (5, 9)	8 (5, 11)	0.622
Laboratory data				
White blood cells (×10^9^/L)	16.9 (12.0, 21.4)	16.5 (13.4, 21.0)	17.1 (11.6, 22.6)	0.733
Neutrophils (×10^9^/L)	14.5 (10.4, 19.7)	14.7 (11.4, 19.3)	14.4 (9.9, 20.1)	0.709
Platelets (×10^9^/L)	183.0 (101.0, 253.5)	196.5 (124.3, 263.3)	172.0 (84.0, 253.0)	0.141
C-reactive protein (mg/L)	120.0 (89.4, 120.0)	120.0 (82.0, 120.0)	120.0 (92.5, 120.0)	0.471
Procalcitonin (ng/mL)	5.6 (2.0, 16.2)	12.7 (3.4, 21.5)	3.5 (1.5, 10.3)	< 0.001
Serum creatinine (μmol/L)	116.7 (66.6, 209.5)	95.1 (58.8, 165.5)	125.6 (70.6, 250.3)	0.007
D-dimer (μg/ml)	1.9 (0.9, 3.8)	2.1 (0.9, 5.0)	1.8 (0.9, 3.3)	0.070
PaO_2_ /FiO_2_	158 (103, 199)	137 (87, 179)	172 (116, 209)	0.002^*^
Mild, n (%)	54 (24.0)	14 (15.6)	40 (29.6)	0.026^#^
Moderate, n (%)	116 (51.6)	48 (53.3)	68 (50.4)	
Severe, n (%)	55 (24.4)	28 (31.1)	27 (20.0)	
Treatments				
Glucocorticoid therapy, n (%)	51 (22.7)	22 (24.4)	29 (21.5)	0.603
Immunoglobulin, n (%)	5 (2.2)	2 (2.2)	3 (2.2)	1.000
Sedative therapy, n (%)	96 (42.7)	50 (55.6)	46 (34.1)	0.001
Vasoactive agent therapy, n (%)	55 (24.4)	25 (27.8)	30 (22.2)	0.342
CRRT, n (%)	33 (14.7)	10 (11.1)	23 (17.0)	0.218
CVC, n (%)	125 (55.6)	55 (61.1)	70 (51.9)	0.171
IMV, n (%)	122 (54.2)	66 (73.3)	56 (41.5)	< 0.001
Length of IMV (days)	3 (2, 7)	3 (2, 6)	3 (2, 8)	0.543
Length of IMV ≥ 3 days, n (%)	80 (65.6)	43 (65.2)	37 (66.1)	0.915
VTE prophylaxis, n (%)	135 (60.0)	50 (55.6)	85 (63.0)	0.267
LMWH, n (%)	108 (48.0)	39 (43.3)	69 (51.1)	0.253
LMWH + physical prophylaxis, n (%)	75 (33.5)	29 (32.6)	46 (34.1)	0.817
Physical prophylaxis only, n (%)	23 (10.3)	9 (10.1)	14 (10.4)	0.950
ICU length of stay (days)	11 (6, 24)	12 (5, 24)	11 (6, 26)	0.563
Hospital length of stay (days)	19 (12, 32)	20 (11, 32)	19 (12, 31)	0.816
Mortality, n (%)	77 (34.2)	45 (50.0)	32 (23.7)	< 0.001

Data are presented as mean ± SD, median (IQR), or n (%). *P* values comparing DVT and non-DVT groups were from a two-sample *t-*test, Mann-Whitney *U* test, χ^2^ test, or Fisher exact test. *P* < 0.05 was considered statistically significant

^*^χ^2^ test comparing DVT and non-DVT groups

^#^χ^2^ test comparing all subcategories

*Abbreviations*: *APACHE* Acute Physiology and Chronic Health Evaluation, *ARDS* acute respiratory distress syndrome, *BMI* body mass index, *CRRT* continuous renal replacement therapy, *CVC* central venous catheterization, *DVT* deep venous thrombosis, *FiO*_2_ fraction of inspired oxygen, *ICU* intensive care unit, *IMV* invasive mechanical ventilation, *IQR* interquartile range, *LMWH* low molecular weight heparin, *mild* 200 mmHg<PaO_2_/FiO_2_ ≤ 300 mmHg, *moderate* 100 mmHg<PaO_2_/FiO_2_ ≤ 200 mmHg, *PaO*_2_ partial pressure of arterial oxygen, *SD* standard deviation, *severe* PaO_2_/FiO_2_ ≤ 100 mmHg, *SOFA* Sequential Organ Failure Assessment, *VTE* venous thromboembolism

### Echocardiographic findings of DVT vs non-DVT patients in overall ARDS cohort

A total of 215 (95.6%) patients received echocardiographic examinations, with 86 patients in the DVT group and 129 patients in the non-DVT group (Table 3). Compared with the non-DVT group, patients with DVT had a lower left ventricular end-systolic volume index (*P* = 0.041) and higher pulmonary artery systolic pressure (*P* = 0.007).

**Table 3:** Echocardiographic Findings of DVT Vs Non-DVT Patients in Overall ARDS Cohort

Variables	Total	DVT	Non-DVT	*P* value
LA diameter (mm)	48 (43, 53)	47 (42, 53)	48 (43, 52)	0.764
LVESVI (mL/m^2^)	46 (43, 49)	46 (42, 48)	47 (44, 49)	0.041
LVEDVI (mL/m^2^)	29 (27, 32)	29 (26, 31)	30 (27, 32)	0.107
Simpson biplane EF (%)	66 (62, 70)	67 (62, 70)	66 (61, 70)	0.513
RA diameter (mm)	45 (41, 48)	45 (41, 48)	44 (40, 48)	0.236
RV diameter (mm)	30 (26, 32)	30 (27, 32)	29 (26, 32)	0.839
PA diameter (mm)	23 (21, 25)	23 (22, 25)	23 (21, 25)	0.933
PASP (mmHg)	40 (36, 48)	43 (38, 60)	38 (35, 46)	0.007
PAH, n (%)	55 (25.6)	25 (29.1)	30 (23.3)	0.338
Pericardial effusion, n (%)	12 (5.6)	4 (4.7)	8 (6.2)	0.628
Tricuspid regurgitation, n (%)	86 (40.0)	38 (44.2)	48 (37.2)	0.306

Data are presented as median (IQR) or n (%). *P* values comparing DVT and non-DVT were from Mann-Whitney *U* test, or χ^2^ test. *P* < 0.05 was considered statistically significant

*Abbreviations*: *ARDS* acute respiratory distress syndrome, *DVT* deep vein thrombosis, *EF* ejection fraction, *LA* left atrial, *LVEDVI* left ventricular end-diastolic volume index, *LVESVI* left ventricular end-systolic volume index, *PA* pulmonary artery, *PAH* pulmonary artery hypertension, *PASP* pulmonary artery systolic pressure, *RA* right atrial, *RV* right ventricular

### Demographic and clinical characteristics of DVT vs non-DVT patients in direct and indirect ARDS cohorts

In the direct and indirect ARDS cohorts (Table 4), patients with DVT were older, had a lower PaO_2_/FiO_2_, a higher level of PCT, and a higher proportion who were given sedative therapy and IMV than patients without DVT (*P* <  0.05 for all). Patients with DVT had lower serum creatinine levels (*P* = 0.003) in the direct ARDS cohort and higher Caprini scores (*P* = 0.021) and higher Padua prediction scores (*P* = 0.008) in the indirect ARDS cohort. More patients with DVT died within 28 days after being diagnosed with ARDS than those without DVT in both groups (*P* = 0.005 and *P* = 0.003, respectively). There were no differences in APACHE II scores and SOFA scores between patients with and without DVT regardless of ARDS subgroup.

**Table 4:** Demographic and Clinical Characteristics of DVT Vs Non-DVT Patients in Direct and Indirect ARDS Cohorts

Characteristics	Direct ARDS (n = 111)			Indirect ARDS (n = 114)		
	DVT (*n* = 44)	Non-DVT (*n* = 67)	*P* *Value*	DVT (*n* = 46)	Non-DVT (*n* = 68)	*P* value
Age (years)	68 ± 11	62 ± 17	0.015	72 ± 15	64 ± 19	0.009
Male, n (%)	32 (72.7)	52 (77.6)	0.557	20 (43.5)	40 (58.8)	0.107
BMI	23.5 (20.6, 25.2)	24.2 (22.2, 27.2)	0.076	24.5 (21.3, 26.8)	24.0 (22.0, 27.3)	0.716
Bedridden time (days)	9 (6, 17)	9 (5, 15)	0.431	10 (4, 19)	7 (4, 14)	0.356
Caprini score	7 (5, 8)	7 (5, 9)	0.891	9 (6, 11)	7 (5,10)	0.021
Padua prediction score	5 (5, 6)	5 (5, 6)	0.689	7 (5, 8)	6 (4, 7)	0.008
APACHE II score	22 (17, 26)	21 (16, 27)	0.568	25 (21, 30)	25 (19, 32)	0.913
SOFA score	6 (5, 9)	6 (4, 10)	0.780	9 (7, 10)	9 (6,11)	0.772
Laboratory data						
White blood cells (×10^9^/L)	14.5 (10.0, 18.3)	14.1 (10.2, 20.7)	0.921	18.4 (15.0, 21.5)	18.1 (13.1, 24.2)	0.630
Neutrophils (×10^9^/L)	12.8 (9.1, 16.8)	12.8 (8.9, 18.0)	0.935	16.1 (13.3, 19.8)	16.1 (11.0, 21.8)	0.669
Platelets (× 10^9^/L)	193.5 (155.3, 279.0)	181.0 (108.0, 257.0)	0.301	202.0 (84.3, 246.5)	148.5 (70.8, 234.0)	0.267
C-reactive protein (mg/L)	120.0 (82, 120.0)	120.0 (89.0,120.0)	0.490	120.0 (86.8, 120.0)	120.0 (94.4, 120.0)	0.758
Procalcitonin (ng/mL)	8.3 (2.6, 17.6)	2.6 (1.1, 9.6)	0.002	15.0 (4.9, 25.0)	4.9 (2.6, 13.5)	0.001
Serum creatinine (μmol/L)	77.3 (56.2, 122.4)	119.0 (70.6, 225.1)	0.003	135.5 (67.3, 208.8)	140.7 (70.0, 275.7)	0.377
D-dimer (μg/ml)	1.8 (0.7, 3.2)	1.2 (0.6, 2.1)	0.211	3.2 (1.6, 7.3)	2.1 (1.3, 4.7)	0.123
PaO2/FiO2	111 (71, 176)	150 (86, 203)	0.035^*^	152 (117, 184)	179 (140, 217)	0.017^*^
Mild, n (%)	5 (11.4)	17 (25.4)	0.062^#^	9 (19.6)	23 (33.8)	0.249^#^
Moderate, n (%)	18 (40.9)	31 (46.3)		30 (65.2)	37 (54.4)	
Severe, n (%) 21 (47.7) 19 (28.4) 7 (15.2) 8 (11.8)	21 (47.7)	19 (28.4)		7 (15.2)	8 (11.8)	
Treatments						
Glucocorticoid therapy, n (%)	16 (36.4)	15 (22.4)	0.108	6 (13.0)	14 (20.6)	0.299
Immunoglobulin, n (%)	1 (2.3)	2 (3.0)	1.000	1 (2.2)	1 (1.5)	1.000
Sedative therapy, n (%)	25 (56.8)	25 (37.3)	0.043	25 (54.3)	21 (30.9)	0.012
Vasoactive agent therapy, n (%)	10 (22.7)	12 (17.9)	0.533	15 (32.6)	18 (26.5)	0.478
CRRT, n (%)	3 (6.8)	9 (13.4)	0.432	7 (15.2)	14 (20.6)	0.468
CVC, n (%)	19 (43.2)	26 (38.8)	0.646	36 (78.3)	44 (64.7)	0.121
IMV, n (%)	34 (77.3)	31 (46.3)	0.001	32 (69.6)	25 (36.8)	0.001
Length of IMV (days)	3 (2, 6)	3 (2, 10)	0.646	3 (2, 7)	3 (2, 8)	0.586
Length of IMV ≥ 3 days, n (%)	21 (61.8)	19 (61.3)	0.969	22 (68.8)	18 (72.0)	0.790
Mortality, n (%)	21 (47.7)	15 (22.4)	0.005	24 (52.2)	17 (25.0)	0.003

Data are presented as mean ± SD, median (IQR), or n (%). *P* values comparing DVT and non-DVT groups were from a two-sample *t-*test, Mann-Whitney *U* test, χ^2^ test, or Fisher exact test. *P* < 0.05 was considered statistically significant

^*^χ^2^ test comparing DVT and non-DVT groups

^#^χ^2^ test comparing all subcategories

*Abbreviations*: *APACHE* Acute Physiology and Chronic Health Evaluation, *ARDS* acute respiratory distress syndrome, *BMI* body mass index, *CRRT* continuous renal replacement therapy, *CVC* central venous catheterization, *DVT* deep venous thrombosis, *FiO*_2_, fraction of inspired oxygen, *ICU* intensive care unit, *IMV* invasive mechanical ventilation, *IQR* interquartile range, *LMWH* low molecular weight heparin, mild 200 mmHg<PaO_2_/FiO_2_ ≤ 300 mmHg, *moderate* 100 mmHg<PaO_2_/FiO_2_ ≤ 200 mmHg, *PaO*_2_ partial pressure of arterial oxygen, *SD* standard deviation, severe, PaO_2_/FiO_2_ ≤ 100 mmHg, *SOFA* Sequential Organ Failure Assessment, *VTE* venous thromboembolism

### Independent predictors of DVT in patients with direct and indirect ARDS

**Fig. 2:**
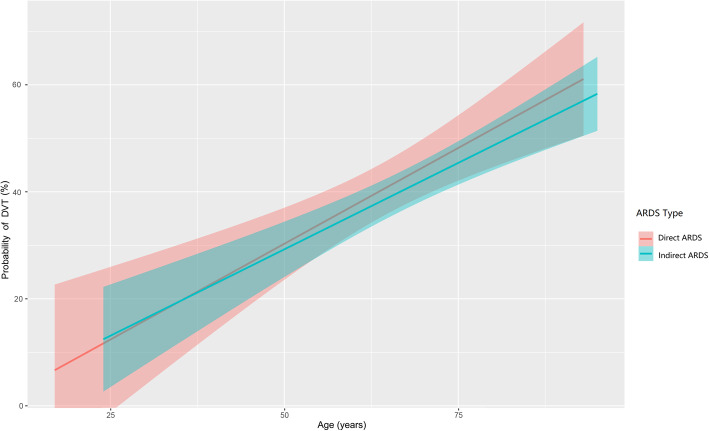
Prevalence of DVT increased with age in patients with ARDS. The prevalence of DVT increased with age in patients with both direct (red line) and indirect ARDS (blue line). However, the occurrence of DVT increased more significantly with increasing age in the direct ARDS than in the indirect ARDS group (test for interaction; *P* = 0.030). Data are adjusted for level of serum creatinine, procalcitonin levels, D- dimer levels, PaO_2_/FiO_2_, and invasive mechanical ventilation. Abbreviations: ARDS, acute respiratory distress syndrome; DVT, deep vein thrombosis; FiO_2_, fraction of inspired oxygen; PaO_2_, partial pressure of arterial oxygen

**Fig. 3:**
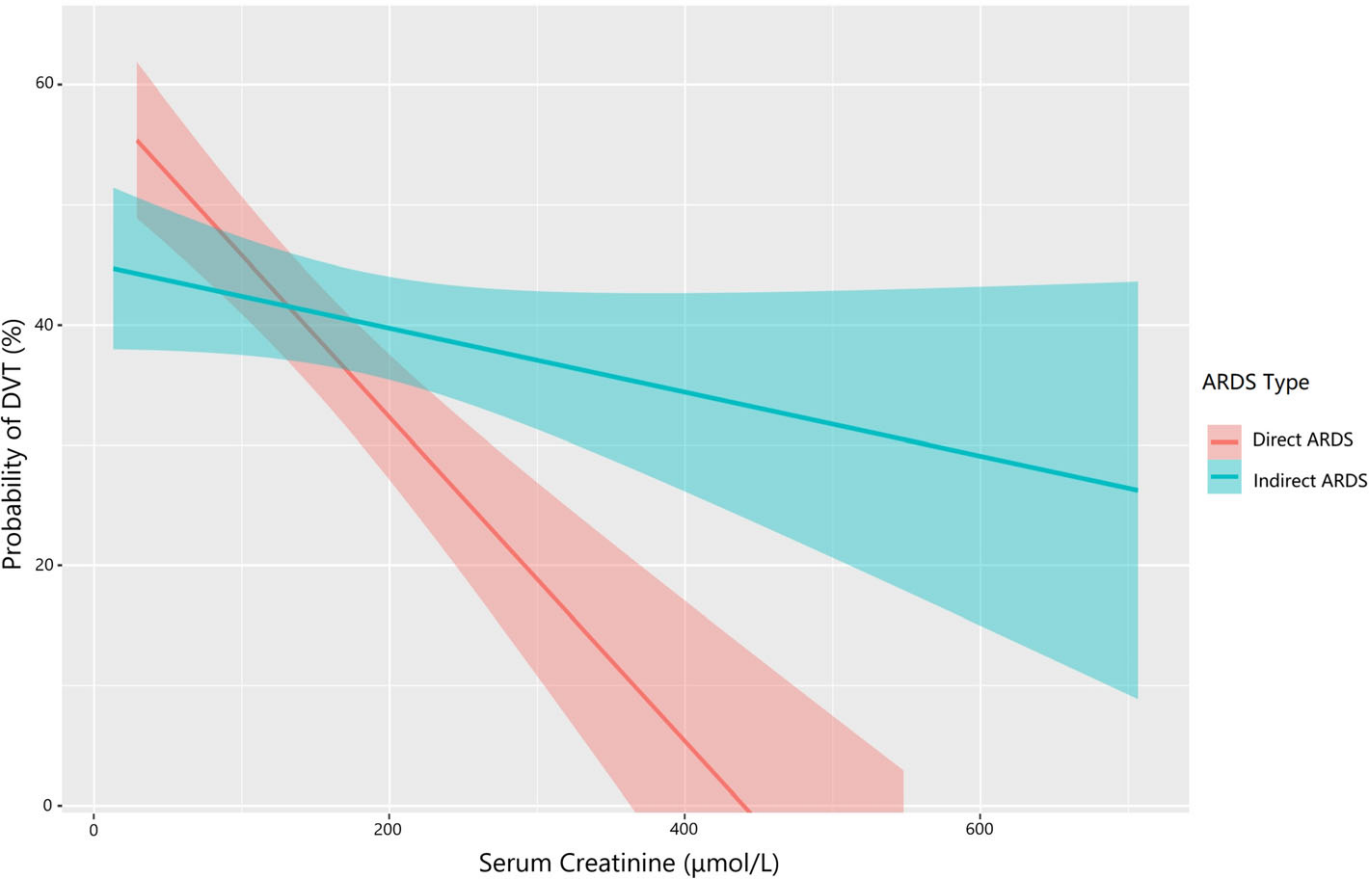
Prevalence of DVT decreased with serum creatinine levels only in the direct ARDS group. The occurrence of DVT in the direct ARDS group (red line) decreased with increasing serum creatinine levels, whereas serum creatinine levels had no association with DVT in the indirect ARDS group (blue line; test for interaction, *P* = 0.006). Data are adjusted for age, procalcitonin levels, D-dimer levels, PaO_2_/FiO_2_, and invasive mechanical ventilation. Abbreviations: ARDS, acute respiratory distress syndrome; DVT, deep vein thrombosis; FiO_2_, fraction of inspired oxygen; PaO_2_, partial pressure of arterial oxygen

Multivariable logistic regression models for DVT were applied in the overall study cohort and then in the direct and indirect ARDS groups, respectively (Table 5). In order to reduce data duplication, we did not include thrombus prediction scores and disease severity scores in the multiple regression models. Because all patients with sedation received IMV, there was a certain degree of overlap between these two variables, so we did not incorporate sedative therapy in the multivariate regression. In the combined and direct ARDS cohorts, age, serum creatinine level, and IMV were independently associated with DVT. In the indirect ARDS group, the independent contributors to DVT were age (*P* = 0.015) and IMV (*P* = 0.024). However, in contrast, the occurrence of DVT increased more significantly with increasing age in those with direct ARDS than in those with indirect ARDS (test for interaction, *P* = 0.030; Fig. 2). Distinct from direct ARDS, the serum creatinine level was not independently associated with increased DVT in the indirect ARDS group (test for interaction, *P* = 0.006; Fig. 3).

**Table 5:** Independent Predictors of Deep Vein Thrombosis in Patients with Direct and Indirect Acute Respiratory Distress Syndrome

Characteristics	Total ARDS (*N* = 225)		Direct ARDS (*N* = 111)		Indirect ARDS (*N* = 114)		*P* Value for Interaction With ARDS Type
	Adjusted OR (95% CI)	*P* value	Adjusted OR (95% CI)	*P* value	Adjusted OR (95% CI)	*P* value	
Age (per 10 years)	1.422 (1.147–1.763)	0.001	1.504 (1.025–2.207)	0.037	1.410 (1.070–1.856)	0.015	0.030
Serum creatinine (per 10 μmol/L)	0.939 (0.908–0.970)	< 0.001	0.857 (0.789–0.930)	< 0.001	0.971 (0.936–1.008)	0.120	0.006
Procalcitonin (ng/mL)	1.033 (0.997–1.070)	0.071	1.035 (0.975–1.099)	0.264	1.035 (0.989–1.084)	0.134	
D-dimer (μg/ml)	1.065 (0.985–1.151)	0.114	1.059 (0.852–1.317)	0.606	1.056 (0.971–1.149)	0.205	
PaO_2_/FiO_2_	0.996 (0.990–1.002)	0.223	0.996 (0.986–1.006)	0.453	0.995 (0.987–1.004)	0.295	
IMV	3.168 (1.579–6.356)	0.001	5.272 (1.536–18.100)	0.008	2.787 (1.144–6.792)	0.024	

Multivariable logistic regression was performed in the overall ARDS cohort and then in the direct ARDS and indirect ARDS groups separately. The interactions of ARDS type (direct or indirect) with age, serum creatinine level, level of procalcitonin, level of D-dimer, PaO_2_/FiO_2_, and IMV were included in the regression analysis

*Abbreviations*: *ARDS* acute respiratory distress syndrome, *CI* confidence interval, *DVT* deep venous thrombosis, *FiO*_2_ fraction of inspired oxygen, *IMV* invasive mechanical ventilation, *OR* odds ratio, *PaO*_2_ partial pressure of arterial oxygen

### Comparison of diagnostic accuracy for screening for DVT of different ROCs in three ARDS cohorts

**Fig. 4:**
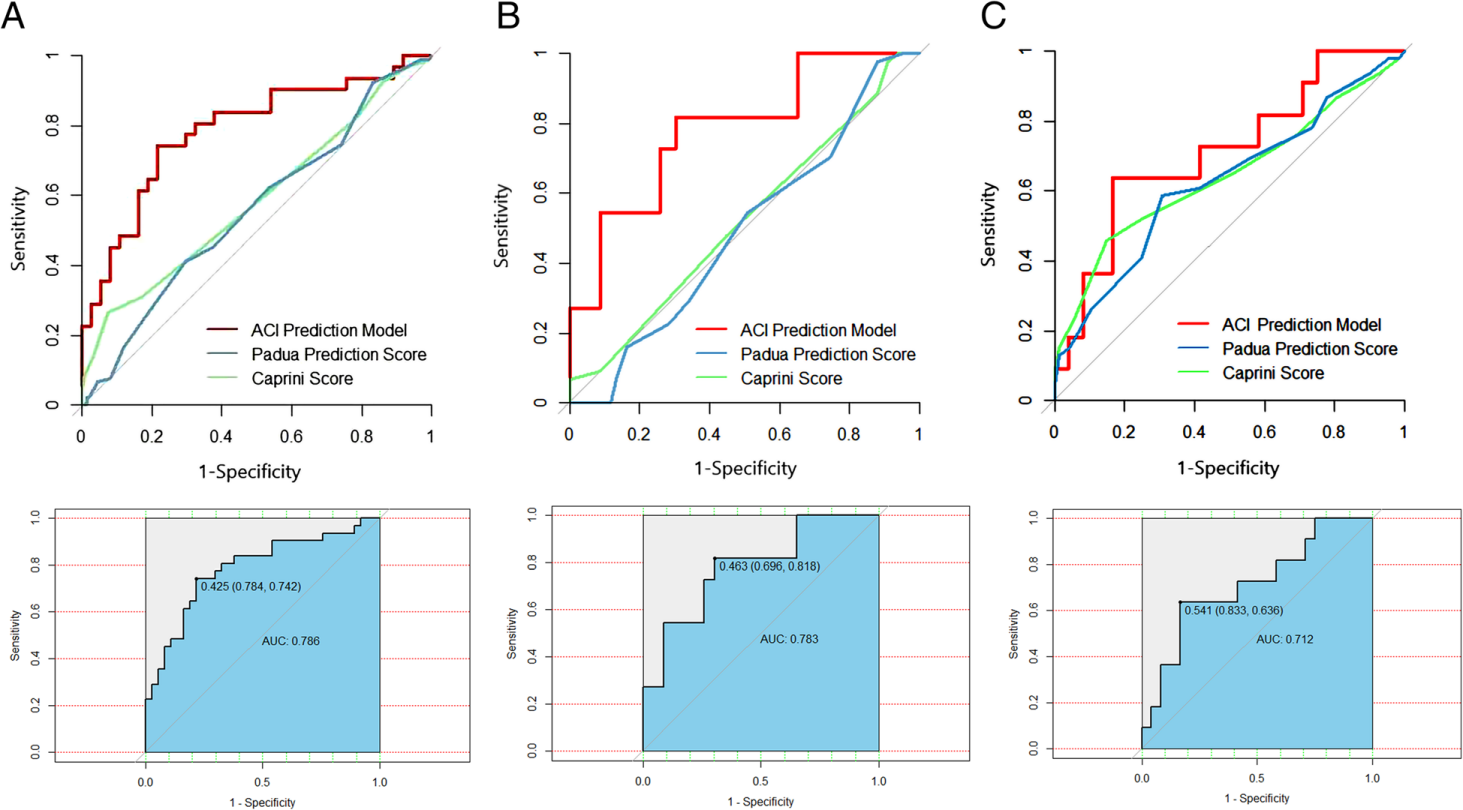
**A**-**C**, Comparison of diagnostic accuracy for screening for DVT of different ROCs in three ARDS cohorts. Patients were split by generating random numbers to produce a training data set (n*0.7) and a validation data set (n*0.3) in the overall, direct, and indirect groups respectively. A, the ACI model which including age, serum creatinine level, and IMV shows satisfactory forecasting ability for DVT (AUC = 0.786; 95% CI: 0.673–0.898; sensitivity: 74.2%; specificity: 78.4%; *P* < 0.001) significantly higher than that of the Padua prediction score (AUC = 0.587; *P* = 0.005 for these two curves) and the Caprini score (AUC = 0.558; *P* = 0.001 for these two curves). B, the ACI model shows a satisfactory ability to predicting DVT (AUC = 0.783; 95% CI: 0.612–0.953; sensitivity: 81.8%; specificity: 69.6%; *P* = 0.004) significantly surpassed the Padua prediction score (AUC = 0.521; *P* = 0.001 for these two curves) and the Caprini score (AUC = 0.492; *P* = 0.006 for these two curves). C, the ACI model shows satisfactory ability for predicting DVT (AUC = 0.712; 95% CI: 0.519–0.905; sensitivity: 63.6%; specificity: 83.3%; *P* = 0.024) has no obvious difference compared with the Padua prediction score (AUC = 0.644; *P* = 0.551 for these two curves) and the Caprini score (AUC = 0.627; *P* = 0.451 for these two curves). Abbreviations: ACI = age + creatinine + IMV; ARDS, acute respiratory distress syndrome; AUC, area under the curve; CI, confidence interval; DVT, deep vein thrombosis; IMV, invasive mechanical ventilation; ROC, receiver operating characteristic

We propose three new ways of combining forecasting models for screening for DVT based on the significant risk factors. The sensitivity and specificity of the corresponding ROC curves of the proposed models were not inferior to those of the Padua prediction score and the Caprini score for screening for DVT (Fig. 4 A - C).

### Survival curves for patients with and without DVT in three ARDS cohorts

**Fig. 5:**
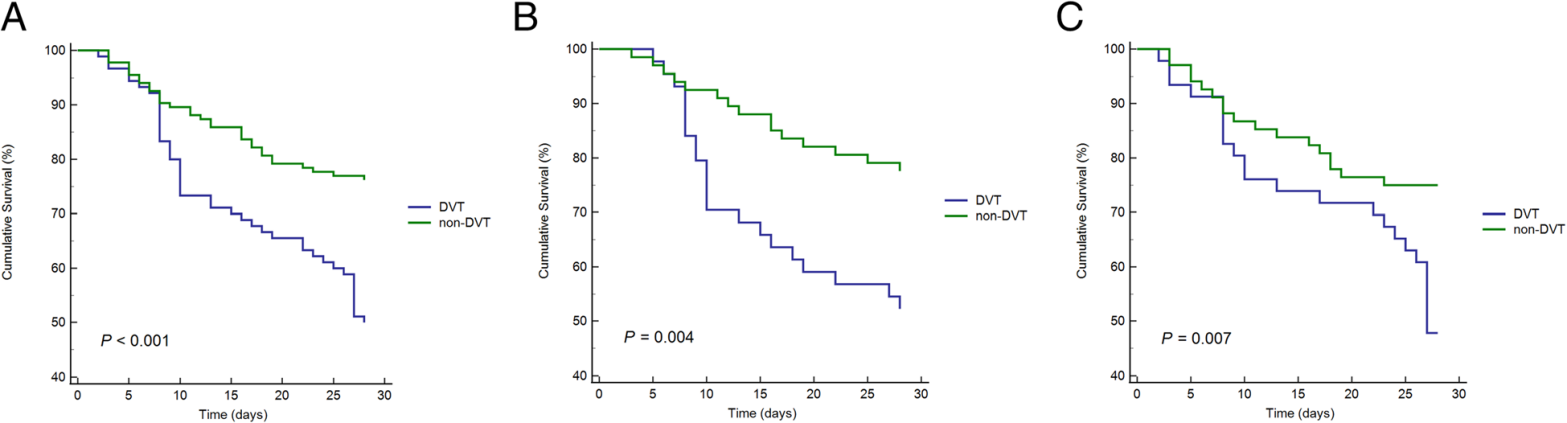
**A**-**C**, Survival curves for patients with and without DVT in the different ARDS cohorts (log-rank test). A, The 28-day survival for patients with and without DVT in the overall ARDS cohort (*P* < 0.001); B, the 28-day survival for patients with and without DVT in the direct ARDS cohort (*P* = 0.004); C, the 28- day survival for patients with and without DVT in the indirect ARDS cohort (*P* = 0.007). Abbreviations: ARDS, acute respiratory distress syndrome; DVT, deep vein thrombosis

Kaplan-Meier survival curves showed that patients with DVT had a significantly lower survival rate within 28 days after ARDS than patients without DVT, not only in the overall ARDS cohort but also in the direct and indirect ARDS groups (*P* <  0.001 [Fig. 5A]; *P* = 0.004 [Fig. 5B]; and *P* = 0.007 [Fig. 5C], respectively).

### The 28-day cumulative incidence rate of DVT in ARDS and non-ARDS patients

**Fig. 6:**
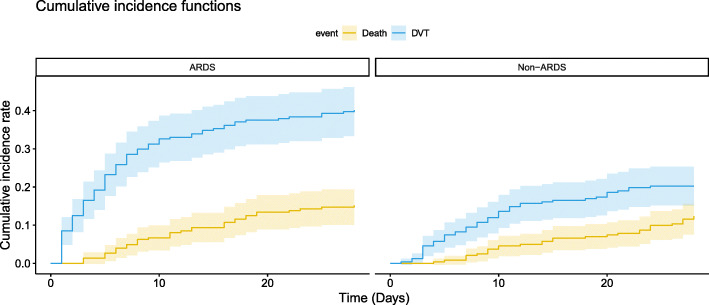
The 28-day cumulative incidence curves of DVT in ICU patients with and without ARDS. Fine-Gray test showed that the 28-day cumulative incidence of DVT and the 28-day mortality in ARDS group were significantly higher than that in non-ARDS group (*P* < 0.001 for both). Abbreviations: ARDS, acute respiratory distress syndrome; DVT, deep vein thrombosis; ICU, intensive care unit

To further explore the incidence rate of DVT in patients with ARDS in ICU, we selected non-ARDS patients (*n* = 266) in ICU during the same period consecutively (with the same exclusion criteria as for the ARDS group) as controls (Fig. 6). The 28-day cumulative incidence rate (95% CI) of DVT in patients with ARDS and non-ARDS were 40.2% (33.8, 46.6%) and 15.2% (10.5, 19.9%) respectively. Fine-Gray test showed that the 28-day cumulative incidence of DVT in ARDS group was significantly higher than that in non-ARDS group (*P* <  0.001). In addition, the 28-day mortality in ARDS group was significantly higher than that in non-ARDS group (*P* <  0.001).

## Discussion

We eventually enrolled 225 patients with ARDS in this study, 111 of whom had direct ARDS and 114 had indirect ARDS. The prevalence of DVT on ultrasound scans in the overall group of patients with ARDS was as high as 40.0%, followed by an undifferentiated prevalence between the cohorts with direct and indirect ARDS (39.6% vs 40.4%, *P* = 0.913). Advanced age, serum creatinine level, and IMV were independently associated with DVT in the overall ARDS group as well as in the direct ARDS cohort. In the indirect ARDS cohort, however, increased DVT was only associated with advanced age and IMV. Patients with DVT had more adverse outcomes than those without DVT, not only in the overall ARDS cohort but also in the direct and indirect ARDS groups.

To the best of our knowledge, this research is the earlier systematic description of DVT in patients with ARDS and of distinct associations among clinical characteristics and DVT in patients with direct and indirect ARDS.

### Prevalence of DVT in patients with ARDS

In 2002, Greets et al. reported that the rates of objectively confirmed DVT in 4 prospective studies ranged from 13 to 31% [27]. In recent years, some research showed that, despite the use of guideline-recommended thromboprophylaxis, the incidence of DVT is still as high as 14 to 37.2% in critically ill patients [2, 3]. Zhang et al. reported that the cumulative incidence of VTE at 7, 14, 21, and 28 days was 4.45, 7.14, 7.53, and 9.55%, respectively, in patients admitted to ICUs in China, even though the patients received guideline-recommended thromboprophylaxis [4]. Several factors probably account for the notably higher prevalence of DVT in our patients. First, most of the previously mentioned studies focused on critically ill patients who were in the ICU for different diseases. ARDS is a more serious type of critical illness that shows an overwhelming systemic inflammatory process accompanied by alveolar epithelial and vascular endothelial injury and an abnormal blood coagulation mechanism associated with significant death and may have a higher risk of DVT. We compared the 28-day cumulative incidence of DVT between ARDS group and non-ARDS group and found a significantly higher incidence of DVT in ARDS group than that in non-ARDS group. Multiple studies have also suggested that the incidence of DVT in patients with ARDS for coronavirus disease 2019 or influenza A (H_1_N_1_) was as high as 42.2 to 85.4% [28–31]. These conditions indicate that direct ARDS itself may be a risk factor for DVT. As the results shown, both direct and indirect ARDS had extremely high incidence rates of DVT. Second, some researchers defined VTE as a pulmonary embolism, proximal DVT, and/or symptomatic distal DVT, thereby excluding asymptomatic isolated distal DVT, which could probably be identified only by screening ultrasound [3]. Furthermore, the heterogeneous patient populations, such as those with different primary conditions, different numbers of days in the hospital, and different preventive measures, may represent a variety incidence.

In recent years, a study of COVID-19 showed a low proportion (14.7%, 23/156) of asymptomatic DVT in a cohort of patients admitted in non-ICUs [32]. The incidence is similar to that reported in other studies about asymptomatic DVT in internal medicine settings and orthopedic surgery settings [33, 34]. Compared with these studies above, the incidence of asymptomatic DVT in ARDS was higher in our study. Also of note is the distal DVT rate (92.2%, 83/90) in patients with ARDS would be significantly higher than that reported in many other hospitalized patients (24.4%, 202/831) [35]. From this article, the conclusions drawn are high proximal DVT or PE recurrent rates (7.9%,16/202) and high mortality (52/202, 25.7%) after isolated distal DVT. So, we ought to pay more attention to the distal DVT in patients with ARDS in order to reduce the mortality.

### Risk factors for DVT in patients with ARDS

Advanced age is a well-recognized risk factor for DVT in hospitalized patients, especially in critically ill patients, which has been included in a variety of thrombosis prevention scoring systems [22, 23]. As expected, in this study, the independent association of increased DVT with advanced age was found in both the direct and indirect ARDS cohorts. Interestingly, however, the contribution of advanced age to DVT differed in the different ARDS cohorts. The prevalence of DVT increased more significantly with advancing age in patients with direct ARDS than in those with indirect ARDS. The reason for this phenomenon may be partly, as previous studies have shown [14, 36] that, in our study, the patients in the indirect ARDS group also displayed more severe disease (higher APACHE II scores and higher SOFA scores) than those in the direct ARDS group, so the effect of advanced age on the overall condition of indirect ARDS was relatively small.

We found an independent association between serum creatinine levels and DVT in our patients. To our knowledge, however, this study earlier assessed the differences in DVT related to renal function in ARDS by direct or indirect etiology. We found that the independent association between the serum creatinine level and the incidence of DVT in ARDS is modified by the underlying ARDS risk factors, with the protective effect on DVT of higher levels of serum creatinine being limited to patients with direct ARDS. However, we did not find a correlation between serum creatinine level and DVT in patients with indirect ARDS, which may be due to the more serious renal impairment and coagulation dysfunction in indirect ARDS, thus weakening the correlation between these two factors. Renal function was associated with dysregulation in coagulation in proportion to the severity of the renal impairment [37]. Some studies have demonstrated that chronic kidney disease and acute kidney injury (AKI) are independent risk factors for VTE [38, 39]. Al-Dorzi et al. pointed out that, for critically ill patients, neither AKI nor end-stage renal disease was an independent risk factor for VTE [40]. McMahon et al. reported that AKI increases the risk for hospitalization-related VTE in a large, heterogeneous population that includes medical and surgical patients. However, this relationship was not seen in patients with traumatic injuries [41]. Some studies have shown that LWMH may have different levels of bioaccumulation in the case of renal insufficiency [42, 43]. The study by Cook et al. indicated that the incidence of DVT for patients with renal insufficiency in ICU who received dalteparin 5000 IU once daily was 5.1% [44], which was far lower than that in the overall population of critically ill patients who received preventive treatment recommended by the guidelines [2–4]. So, we speculate that the same dose of LWMH may play a stronger role in the prevention of DVT in the case of renal insufficiency. Unfortunately, due to the retrospective nature of the study, the decrease of LWMH metabolism in patients with AKI and higher level of serum creatinine was based on the conjecture of clinical data analysis, and we did not detect the activity of anti-factor Xa.

ARDS is a clinical syndrome with high mortality manifested by severe acute hypoxemia, which usually requires MV, especially IMV [8]. With IMV, sedation and immobilization are often performed simultaneously, which would aggravate blood stasis and increase the risk of DVT. Some studies have shown that IMV is a high-risk factor for DVT [29, 45]. Knudso et al. pointed out that IMV administered for more than 3 days is an independent risk factor for VTE [46]. As the duration of IMV increased, the risk of DVT increased [3]. Our research showed that both IMV and sedation were risk factors for DVT. Because all sedated patients in our study were treated with IMV, we only included IMV in the multivariate regression analysis. The results showed that IMV was an independent risk factor for DVT in both direct and indirect ARDS cohorts. However, in this study, compared with patients in the non-DVT group, the duration of IMV in the DVT group did not increase significantly, possibly because our small number of cases resulted in no statistically significant difference.

In direct and indirect ARDS cohorts, neither the APACHE II score nor the SOFA score was associated with the occurrence of DVT, presumably because the serum creatinine level, which was negatively correlated with the occurrence of DVT, was included in these two scoring systems [24, 25], thus weakening the correlation between severity scores and DVT.

### Different ROCs for screening for DVT in ARDS cohorts

Differences in predictors of DVT between direct and indirect ARDS partly support the growing body of literature suggesting that there are subphenotypes of ARDS that affect clinical outcomes [12–14, 16]. Our results suggest that subgroup analyses of ARDS are probably beneficial for stratifying and predicting the risk of DVT. We used age, IMV, and serum creatinine levels to predict DVT in the overall and the direct and indirect ARDS cohorts, respectively, and found that, in ARDS, the combined application of these indicators was not inferior to the current commonly used thrombus prediction scores, such as the Padua prediction score [23] and the Caprini score [22], for screening for DVT. Especially for direct ARDS, the combination of age, IMV, and serum creatinine level yielded a sensitivity of 81.8% and a specificity of 69.6% for scanning for DVT. A possible reason is that the Padua prediction score and the Caprini score apply to the general medical and surgical patients in the hospital. As a serious clinical pathophysiological syndrome with an overwhelming inflammatory response and coagulation abnormalities, ARDS has unique clinical characteristics and serious complications. The predictive value of the commonly used thrombus prediction method may be limited to screening for DVT in a patient with a critical illness such as ARDS.

### Prognosis of DVT in patients with ARDS

Similar to the results of some previous studies [30, 47, 48], our results showed that DVT was associated with adverse outcomes in all the ARDS cohorts. Although there was no significant difference between length of stay in hospital and length of stay in ICU, Kaplan-Meier curves showed that the 28-day survival rate of patients with DVT was significantly lower than that of patients without DVT in all the ARDS cohorts. To validate the prognosis of DVT in patients with ARDS, we further plotted 28-day cumulative incidence curve of DVT, with death as the competitive risk, and found that the mortality increased with increasing incidence of DVT. The relationship between inflammation and thrombosis has been identified in different clinical scenarios where the inflammatory process and coagulation abnormalities are clearly interlinked [49, 50]. The high incidence of DVT in ARDS may be a manifestation of a severe inflammatory response with significant coagulation and fibrinolytic dysfunction [50]. In addition, there is a 50% chance for patients with untreated proximal DVT to develop symptomatic PE within 3 months [51]. PE might aggravate the hypoxemia of ARDS patients and then result in lower actuarial survival rates. If there was any clinical suspicion of PE, a CTPA would be considered and obtained, if possible. Unfortunately, due to the critical condition of ARDS patients, CTPA examination was restricted. We only underwent CTPA examination on 3 patients with highly suspected PE and diagnosed with PE, which significantly underestimated the incidence of PE. The presence of PE associated with DVT may also be a cause of poor survival in patients with DVT.

Our study has some limitations. First, our sample size was small, which may underestimate the influence on DVT of factors such as obesity, being bedridden, and the insertion of a central venous catheter. Second, some patients had ultrasound scans only in the early stage of ARDS and did not have continuous dynamic monitoring, which may cause the incidence of DVT to be underestimated. Third, due to the critical condition of patients with ARDS, CTPA examinations were restricted. We performed CTPA examinations on only 3 patients with a high suspicion of PE and then confirmed the diagnosis of PE, which significantly underestimated the incidence of PE. Finally, this study is a retrospective study. We hope to conduct a prospective larger cohort to further clarify the incidence of DVT in patients with different subtypes of ARDS, to determine the corresponding risk factors, and to explore optimized individualized preventive measures in the case of ARDS to reduce DVT-related adverse prognoses.

## Conclusions

The incidence of DVT is extremely high in patients with ARDS and may be associated with adverse outcomes. The risk factors for DVT are age, serum creatinine level, and IMV in ARDS. We suspect that DVT is probably an additional risk factor for the death of ARDS in hospitalized patients. The classification and analysis of ARDS may help to provide more accurate screening for DVT and risk stratification and lead to corresponding measures to improve the clinical outcome of patients with ARDS.

## Data Availability

All data analyzed during the study are presented in the main manuscript. The anonymous dataset is available from the corresponding author upon reasonable request.

## Abbreviations

AKI: Acute kidney injury
APACHE: Acute Physiology and Chronic Health Evaluation
ARDS: Acute respiratory distress syndrome
AUC: Area under the curve
CI: Confidence interval
CTPA: Computed tomography pulmonary angiography
DVT: Deep vein thrombosis
FiO_2_: Fraction of inspired oxygen
ICU: Intensive care unit
IMV: Invasive mechanical ventilation
IQR: Interquartile range
MV: Mechanical ventilation
OR: Odds ratio
PaO_2_: Partial pressure of arterial oxygen
PCT: Procalcitonin
PE: Pulmonary embolism
ROC: Receiver operating characteristic
SD: Standard deviation
SOFA: Sequential Organ Failure Assessment
VTE: Venous thromboembolism
